# Trends in the epidemiology of urinary tract infections in pregnancy at a tertiary hospital in Johannesburg: Are contemporary treatment recommendations appropriate?

**DOI:** 10.4102/sajid.v36i1.328

**Published:** 2021-12-09

**Authors:** Trusha Nana, Shastra Bhoora, Vindana Chibabhai

**Affiliations:** 1 Department of Clinical Microbiology and Infectious Diseases, University of the Witwatersrand, Johannesburg, South Africa; 2 Microbiology Laboratory, Charlotte Maxeke Johannesburg Academic Hospital, National Health Laboratory Services, Johannesburg, South Africa; 3 Department of Obstetrics and Gynaecology, University of the Witwatersrand, Johannesburg, South Africa; 4 Department of Obstetrics and Gynaecology, Charlotte Maxeke Johannesburg Academic Hospital, South Africa; 5 Gauteng Department of Health, Johannesburg, South Africa; 6 Department of Obstetrics and Gynaecology, Faculty of Medicine, Baylor College, Houston, United States of America

**Keywords:** urinary tract infections, asymptomatic bacteriuria, pregnancy, antimicrobial resistance, surveillance, trends

## Abstract

**Background:**

Urinary tract infections (UTIs) are common during pregnancy and are associated with maternal and foetal complications. Empiric antibiotic choices in pregnancy require consideration of efficacy and safety, resulting in limited oral options. With rapidly evolving antibiotic resistance, surveillance to guide empiric treatment recommendations is essential.

**Methods:**

A retrospective analysis of urine culture isolates from the Charlotte Maxeke Johannesburg Academic Hospital (CMJAH) Obstetrics Department for 1 January 2015 to 31 December 2020 was performed.

**Results:**

The top 3 pathogens were *Escherichia coli, Enterococcus faecalis* and *Klebsiella pneumoniae*. For *E. coli* susceptibility to cefuroxime declined (95% to 81%, *p* < 0.0001). Similarly, the *E. coli* extended spectrum beta-lactamase rate increased from 5% to 10% (*p* = 0.04). *E. coli* susceptibility to nitrofurantoin (93%) and fosfomycin (96%) remained high. In 2019, carbapenem-resistant *K. pneumoniae* emerged. Ampicillin susceptibility was high amongst the *E. faecalis* isolates. Amoxicillin-clavulanate demonstrated high levels of activity against the top 3 uropathogens.

**Conclusion:**

The Essential Drug List recommended antibiotics for lower UTIs, nitrofurantoin and fosfomycin, are appropriate empiric options for *E. coli*, the most common uropathogen in the CMJAH obstetric population. The high rate of *E. faecalis* susceptibility to nitrofurantoin reported from other Gauteng tertiary obstetric patients, suggests that nitrofurantoin will provide adequate empiric cover for a large proportion of UTIs. However, the determination of the *E. faecalis* nitrofurantoin and fosfomycin susceptibility rates in the CMJAH obstetric population will provide useful data. Periodic surveillance at the various levels of antenatal care in different regions of South Africa and the determination of risk factors for infections with resistant uropathogens is needed.

## Background

Urinary tract infections (UTIs) are common during pregnancy. The hormonal and anatomical changes that occur during each trimester render pregnant women vulnerable to UTIs. In the first trimester, progesterone promotes smooth muscle relaxation and the ureters begin to dilate. Towards the mid-second trimester, the growing gravid uterus compresses the urogenital system, thus worsening hydronephrosis, urinary stasis and utero-vesical reflux. These collective adaptations favour asymptomatic and symptomatic UTIs.^1^ Asymptomatic bacteriuria (ASB), defined as the presence of ≥ 10^5^ colony forming units (CFUs)/mL of a bacterial species in a voided urine specimen in the absence of UTI symptoms or signs, is most common.^2^ Asymptomatic bacteriuria occurs in 2% – 15% of pregnancies and is associated with an increased risk of preterm labour.^1,2,3,4^ Screening for and treatment of ASB are recommended early in pregnancy.^2,3^ Treatment of ASB decreases the risk of pyelonephritis and may also reduce premature birth and very low birth weight deliveries.^2^ Symptomatic UTIs, cystitis and pyelonephritis complicate 1% – 2% of pregnancies and are associated with several maternal and foetal complications.^1,4^

Current epidemiologic data are required to guide empiric antibiotic choices. Monitoring of pathogen spectrum and antibiotic susceptibility patterns is paramount in an era of rapidly developing antibiotic resistance. A 2011 study evaluating the epidemiology of community-acquired UTIs in women presenting to one public and four private primary healthcare centres in Gauteng reported high rates of pathogen susceptibility to fosfomycin (95.5%) and nitrofurantoin (91%) and lower rates of susceptibility to amoxicillin-clavulanate (82.8%) and co-trimoxazole (44.3%).^5^ A multicentre KwaZulu-Natal (KZN) obstetric department study found a decline in susceptibility to commonly used beta-lactam antibiotics between 2015 and 2019 amongst urinary *Escherichia coli* isolates.^6^

Antibiotic choices for the empiric treatment of UTIs in pregnancy must take into consideration both the efficacy and safety of the antimicrobial agents, with oral formulations preferred for outpatient treatment of UTIs. An additional real-world factor influencing empiric choices is the availability of specific drugs at individual healthcare centres. The South African Essential Drug List (EDL) currently recommends the use of empiric nitrofurantoin 100 mg 6 hourly for 5 days or a single 3 g dose of fosfomycin for treatment of cystitis in pregnancy. For pyelonephritis, empiric therapy with intravenous ceftriaxone is recommended.^7^ Nitrofurantoin and fosfomycin cannot be used for the treatment of pyelonephritis.

The worldwide problem of antimicrobial resistance (AMR) has led to the World Health Organization’s publishing of the priority pathogens list, as well as the global action plan on AMR. Improving the understanding of AMR, strengthening knowledge through surveillance and optimising the use of antimicrobials are some of the key objectives of these initiatives.^8,9^ The obstetric population is a vulnerable patient population. Severe infections and inadequately treated infections can result in poor outcomes not only to the mother but also to the unborn infant. Application of these principles to this patient population is highly relevant.

The objective of the study was to analyse the prevalence and antimicrobial susceptibility profiles of uropathogens in the obstetric patient population at the Charlotte Maxeke Johannesburg Academic Hospital (CMJAH) over 6 years, with the aim of providing guidance on empiric therapy and recommendations for ongoing surveillance.

## Methods

### Study setting and patient population

The CMJAH is a 1088 bed tertiary facility located in Johannesburg, South Africa. The hospital provides numerous specialist services. This includes the obstetric department, which provides antenatal care services to ‘walk-in’ patients and high-risk patients (for whom basic antenatal care is inadequate) referred from the surrounding healthcare centres. The obstetric department has a 28-bedded high-risk antenatal ward and a five-bedded high-care unit. Antenatal services are delivered from Monday to Friday, whilst emergency obstetric services are available 24 h in the maternity admissions ward located near the main casualty of the hospital. The yearly average of antenatal clinic (ANC) visits and deliveries in the department is 21 000 and 8850, respectively.

### Study design

We performed a retrospective laboratory analysis of positive urine culture isolates from three areas of the CMJAH obstetric department: the antenatal clinic, maternity admissions and labour ward from 1 January 2015 to 31 December 2020.

### Investigation of urinary tract infection in pregnancy

Midstream urine (MSU) samples are submitted for cell count, culture and susceptibility testing for patients with UTI symptoms and when urine dipsticks, which are routinely performed at each ANC visit, detect the presence of leucocyte esterase or nitrites. For maternity admissions and labour ward, MSU or samples from catheters in catheterised patients are submitted when the urine dipsticks show leucocyte esterase or nitrites or a septic-work-up is performed. In labour ward, samples are also submitted from patients with significant proteinuria.

### Laboratory testing

Samples were transported to the on-site CMJAH National Health Laboratory Services (NHLS) Microbiology laboratory, which operates 24-h a day. Samples were processed using standard microbiological techniques. Organism identification and antimicrobial susceptibility testing (AST) were performed on automated systems (Vitek® 2 and Vitek® MS, bioMerieux, France) and with manual biochemical and Kirby Bauer disk diffusion methods. Disk diffusion was used for fosfomycin susceptibility testing of *E. coli* isolates. During the study period, fosfomycin and nitrofurantoin testing were not performed on *Enterococcus faecalis* isolates as these antimicrobials are not present on the Vitek® AST panel used for Gram-positive organisms.

### Data collection

Data for positive urine cultures submitted from CMJAH for the study period were extracted from the NHLS laboratory information system, TrakCare (InterSystems, USA).

### Data analysis

The data extract was filtered to exclude cultures from areas other than the three relevant obstetric ones. Data were deduplicated to include only the first isolate per species per patient. Non-speciated coagulase-negative staphylococci (CoNS), non-saprophyticus species of CoNS, other bacterial contaminants and *Candida* species were removed from the data set.^10^

Interpretation of AST results was performed using the Clinical and Laboratory Standards Institute (CLSI) M100 guideline.^11^ For all antibiotics (including the beta-lactams), intermediately susceptible results were reported as resistant. Antimicrobial susceptibility testing results were reported when 30 or more isolates per species were present for that period. The CLSI analysis and presentation of cumulative AST test data M39-A4 guideline was used.^12^

### Definitions

Extended spectrum beta-lactamase (ESBL): Non-susceptibility to one or more third-generation cephalosporins was used as a marker of ESBL production.

Carbapenem-resistant Enterobacterales (CRE): Non-susceptibility to one or more carbapenems was used as a marker of carbapenem resistance.

### Statistical analysis

Data analysis was performed using Microsoft Excel and EpiTools epidemiological calculators.

Categorical data were reported as proportions and percentages with 95% confidence interval (CI). Where AST results were not available for a specific pathogen–antibiotic combination, the denominators for percentage susceptibility calculations were adjusted to reflect this. The Z-proportions test was used to analyse differences between pathogen prevalence and susceptibility rates. Two-tailed *p*-values were reported with values < 0.05 considered statistically significant.

### Ethical considerations

An ethics waiver was obtained from the University of the Witwatersrand Human Research Ethics Committee, waiver number: W-CBP-210122-01.

## Results

**FIGURE 1:**
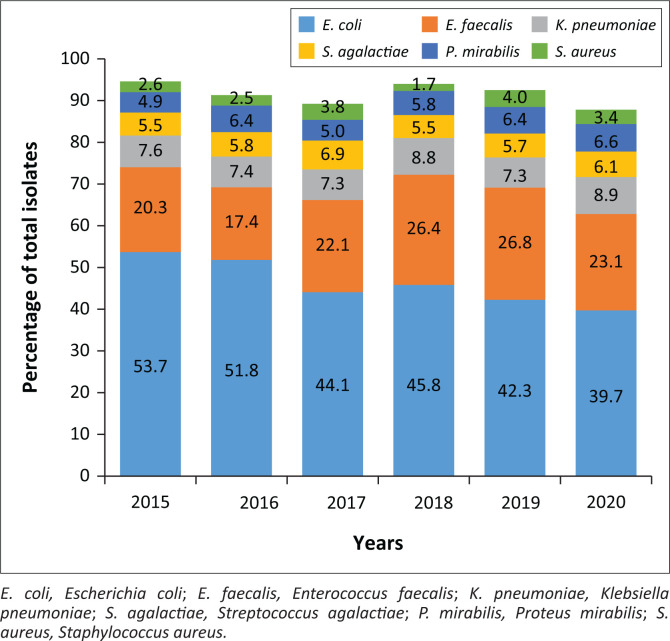
Yearly distribution of top six uropathogens.

Following removal of common commensals, the total number of deduplicated positive culture results was 4739, with a yearly breakdown of 344 (2015), 517 (2016), 755 (2017), 998 (2018), 1151 (2019) and 974 (2020). Figure 1 illustrates the top six pathogens per year. *Escherichia coli* was predominant, followed by *E. faecalis* across all time periods. In 2020, these two organisms were the causative pathogens in 62.8% (95% CI [59.8–65.8]) of the UTIs. The top three pathogens (including *Klebsiella pneumoniae*) accounted for 71.7% (95% CI [68.9–74.5]) of all infections in 2020. *Streptococcus agalactiae, Proteus mirabilis* and *Staphylococcus aureus* were responsible for most of the remaining infections. Less commonly isolated species included *Enterobacter* spp., *Citrobacter* spp. and *Staphylococcus saprophyticus*. The prevalence of *E. coli* declined significantly over the study period (2015: 53.7%, 95% CI [48.5–58.9] and 2020: 39.7%, 95% CI [36.7–42.7], *p* < 0.0001).

The susceptibility results for the common Gram-negative pathogens are summarized in Table 1. Susceptibility to ampicillin was low amongst these Gram negatives. Similarly, *E. coli* exhibited low susceptibility rates to cotrimoxazole. Amoxicillin-clavulanate and cefuroxime showed similar rates of activity against the respective Gram-negative pathogens. The pooled 2019/2020 *E. coli* amoxicillin-clavulanate data demonstrated a susceptibility rate of 82% (95% CI [79.1–84.2]). The susceptibility of *E. coli* to cefuroxime declined from 95% (95% CI [90.7–95.2]) in 2015 to 81% (95% CI [76.4–84.5], *p* < 0.0001) in 2020. The *E. coli* ESBL rate increased from 5% (95% CI [2.5–9.1]) in 2015 to 10% (95% CI [7.2–13.2], *p* = 0.04) in 2020. Both nitrofurantoin and fosfomycin retained high rates of activity against *E. coli. E. coli* susceptibility to these two agents remained stable over the study period (nitrofurantoin 2015: 95%, 95% CI [90.3–97.4] vs. 2020: 93%, 95% CI [90.3–95.5], *p* = 0.359 and fosfomycin 2015: 97%, 95% CI [90.1–99.3] vs. 2020: 96%, 95% CI [93.8–98.1], *p* = 0.552). Of the 38 ESBL-producing *E. coli* isolates in 2020, 92% (95% CI [78.5–98]), 94% (95% CI [81.8–99.5]) and 47% (95% CI [32.5–62.7]) were susceptible to nitrofurantoin, fosfomycin and amoxicillin-clavulanate, respectively. The emergence of carbapenem-resistant *K. pneumoniae* in this patient population occurred in 2019 (3/84, 4%, 95% CI [0.8–10.6]). Nitrofurantoin susceptibility rates *in K. pneumoniae* were consistently low (38% – 46%).

**TABLE 1:** Antibiotic susceptibility results of common gram-negative uropathogens.

Organisms	Year	Number of isolates	Percentage susceptibility (95% Confidence interval)								Resistance (95% CI)	
			Amoxicillin	Ampicillin clavulanate	Cefuroxime	Ceftriaxone	Ertapenem	Cotrimoxazole	Nitrofurantoin	Fosfomycin	%ESBL	%CRE
*E. coli*	2015	185	24 (18.4–30)	88 (82.4–92)	95 (90.7–95.2)	95 (90.9–97.5)	100 (97.6–100)	37 (30.1–43.9)	95 (90.3–97.4)	97 (90.1–99.3)	5 (2.5–9.1)	0
	2016	268	32 (26.3–37.4)	79 (74.2–83.9)	91 (94.2–98.8)	95 (91.2–96.9)	100 (97.5–100)	37 (30–43.9)	93 (87.6–95.7)	†	5 (3.1–8.8)	0
	2017	346	30 (25.1–34.7)	81 (76.1–84.4)	86 (81.6–89.4)	90 (85.8–92.4)	100 (98.7–100)	35 (30.2–40.4)	93 (90.1–95.7)	98 (94.8–99.2)	7.6–14.2)	0
	2018	422	27 (23.1–31.5)	75 (70.6–78.9)	81 (76.8–84.5)	91 (87.6–93.2)	100 (98.9–100)	34 (29.9–39)	94 (90.6–95.6)	98 (95.6–98.9)	9 (6.8–12.4)	0
	2019	488	33 (28.9–37.2)	79 (75.6–82.8)	81 (77.6–84.6)	93 (90.4–95)	100 (99.1–100)	39 (34.9–43.6)	93 (90.1–94.8)	98 (96.2–99.3)	7 (5–9.6)	0
	2020	387	36 (31.1–40.7)	85 (81.1–88.2)	81 (76.4–84.5)	90 (86.8–92.8)	100 (98.7–100)	44 (39.4–49.3)	93 (90.3–95.5)	96 (93.8–98.1)	10 (7.2–13.2)	0
*K. pneumoniae*	2015	27										
	2016	38	0	79 (63.4–89.2)	89 (75.3–96.4)	92 (78.5–98)	100 (86.5–100)	†	41 (26.3–56.5)	-	8 (2–21.5)	0
	2017	55	0	85 (73.6–92.7)	87 (75.7–94)	89 (77.8–95.3)	100 (92.2–100)	84 (71.5–91.4)	39 (27–52.2)	-	11 (4.7–22.2)	0
	2018	88	0	80 (69.9–86.7)	85 (75.4–91)	85 (76.2–91.3)	100 (95–100)	72 (61.8–80.5)	38 (28.4–48.4)	-	15 (8.7–23.8)	0
	2019	84	0	79 (68.6–86.1)	84 (74.3–90.5)	86 (76.5–91.8)	96 (89.4–99.2)	63 (52.4–72.6)	43 (32.3–53.4)	-	15 (8.2–23.5)	4 (0.8–10.6)
	2020	87	0	80 (70.2–87.2)	84 (74.9–90.8)	87 (78.1–92.8)	99 (93–100)	80 (69.9–87.1)	46 (35.3–56.5)	-	13 (7.2–21.9)	1 (0.4–7)
*P. mirabilis*	2015	17										
	2016	33	39 (24.6–56.4)	91 (75–97.5)	82 (65.2–91.8)	97 (83.3–100)	100 (87.6–100)	†	0	-	3 (0–16.7)	0
	2017	38	63 (47.2–76.7)	92 (78.5–98)	97 (84.9–100)	97 (85.3–100)	100 (89.1–100)	71 (55.1–83.1)	0	-	3 (0–14.7)	0
	2018	58	55 (42.4–67.3)	91 (80.3–96.5)	96 (87.4–99.7)	98 (90–100)	100 (92.4–100)	52 (39.2–64.1)	0	-	2 (0–10)	0
	2019	74	51 (40.2–62.4)	97 (89.8–99.8)	96 (87.8–99)	96 (88.3–99.1)	99 (91.9–100)	45 (33.8–55.9)	0	-	4 (0.9–11.7)	1 (0–8.1)
	2020	64	57 (44.9–69)	95 (85.1–98.8)	97 (88–99.7)	97 (88.2–99.8)	100 (92.7–100)	54 (41.7–66)	0	-	3 (0.2–11.8)	0

ESBL, extended spectrum beta-lactamase; CRE, carbapenem-resistant enterobacterales; *E. coli, Escherichia coli; K. pneumoniae, Klebsiella pneumoniae; P. mirabilis, Proteus mirabilis*.

† , Less than 30 susceptibility results available.

All *E. faecalis* (except for 1/90 isolates in 2016) and *S. agalactiae* isolates were susceptible to ampicillin. *S. aureus* susceptibility to cloxacillin in 2019 and 2020 was 78% (95% CI [64.2–87.9]) and 85% (95% CI [68.6–93.8]), respectively, (*p* > 0.05).

In 2020, the cumulative coverage provided by amoxicillin-clavulanate for the top two and three pathogens, which comprised 62.8% and 71.7% of total isolates, was 89% (95% CI [86.5–91.5]) (Table 2) and 88% (95% CI [85.6–90.4]), respectively. Cumulative coverage rates provided by nitrofurantoin and fosfomycin were not determined because of the lack of data for *E. faecalis*.

**TABLE 2:** Cumulative coverage for top uropathogens.

Pathogens	Number of isolates		Antibiotic susceptibility (%)					
	*n*	%	Ampicillin (%)	Amoxicillin-clavulanate (%)	Cefuroxime (%)	Nitrofurantoin (%)	Cotrimoxazole (%)	Fosfomycin (%)
*E.coli*	387	39.7	36	82†	81	93	44	96
*E.faecalis*	225	23.1	100	100	0	‡	0	‡
**Top 2 pathogens cumulative susceptibility**	612	62.8	59	89	51	-	28	-
*K. pneumoniae*	87	8.9	0	80	84	46	80	0
**Top 3 pathogens cumulative susceptibility**	699	71.7	52	88	55	-	34	-

*E. coli, Escherichia coli; E. faecalis, Enterococcus faecalis; K. pneumoniae, Klebsiella pneumoniae*.

† , Average of pooled 2019/2020 *E. coli* amoxicillin-clavulanate data.

‡ , Susceptibility data not available.

## Discussion

The top three pathogens (in descending order: *E. coli, E. faecalis* and *K. pneumoniae*) remained consistent from 2015 to 2020. Whilst the high *E. coli* susceptibility rates to nitrofurantoin and fosfomycin remained stable, a significant increase in ESBLs was seen. Similarly, the ESBL rate and emergence of carbapenem resistance in the *K. pneumoniae* isolates is problematic.

There is a lack of national surveillance at the antenatal care and primary healthcare-level in general, where empiric therapy for UTIs is frequently prescribed. However, contemporary single centre and regional studies within South Africa can provide useful data to validate current recommendations, to signal when these recommendations need to be modified and to identify gaps in epidemiological data that need to be addressed. Regional or local changes in pathogen spectrum and proportions and their associated antibiotic susceptibility patterns may also mandate changes in recommendations in local guidelines. When resistance rate to a specific antibiotic exceeds 20%, this antibiotic is no longer suitable for use as an empiric therapy choice.^13^ Beta-lactam antibiotics are renally excreted and reach high urinary concentrations. The European Committee of Antimicrobial Susceptibility Testing (EUCAST) and CLSI antibiotic interpretive breakpoints reflect this with specific (higher) breakpoints for uncomplicated UTIs and comments for the intermediately susceptible breakpoints, respectively. Laboratories must report AST results for UTIs accordingly.

The common pathogens identified in this study are in general consistent with the findings of other recently published South African studies.^6,14,15^ The relatively high number of *E. faecalis* infections (2016: 17.4%) in this study is similar to that seen in the pooled 2015–2019 data for four Gauteng tertiary centres (including CMJAH) but higher than that reported for 2011–2016 in KZN (4.2%). The second most common pathogen in KZN was *K. pneumoniae*. The KZN study included *Candida* species, but this is unlikely to fully account for the differences. Limited Gauteng data from 2011 showed *E. faecalis*, whilst being the predominant Gram-positive pathogen, comprised only 4% of all pathogens.^5^ The reason(s) for the higher *E. faecalis* and declining *E. coli* rates in this patient population are not well understood. The *E. coli* rates were lower than those in the given cited studies: 54.2% (Zwane et al.), 56% (Bhola et al.) and 56% (Lewis et al.). Comparison of centre-specific data (with CMJAH, Chris Hani Baragwanath Academic Hospital and Rahima Moosa Mother and Child Hospital based in Johannesburg and Steve Biko Academic Hospital in Tshwane) from the Zwane et al. publication may yield useful information regarding trends in *E. coli* rates in Gauteng obstetric patient populations.

The high rate of resistance to ampicillin and cotrimoxazole amongst the Enterobacterales in this study and globally precludes the use of these antibiotics for empiric therapy of UTI in the pregnant patient.^16^ The significant decline in *E. coli* susceptibility to cefuroxime coupled with the intrinsic resistance of enterococci to cephalosporins reduces the utility of empiric cefuroxime use at CMJAH and other settings with a significant burden of *E. faecalis* UTIs. *Escherichia coli* cefuroxime susceptibility in KZN hospitals was similarly low at 76%.^6^ Amongst the *E. coli* isolates, amoxicillin-clavulanate susceptibility was similar to cefuroxime. However, amoxicillin-clavulanate is active against *E. faecalis* and thus provides superior cover when used empirically at CMJAH. *Escherichia coli* amoxicillin-clavulanate susceptibility reported by Zwane et al. and Bhola et al. (82% and 78.4%, respectively) was comparable to the 2019/2020 CMJAH rate of 82%.

The South African EDL recommends ceftriaxone as empiric therapy for pyelonephritis in pregnancy.^7^ The increase in *E. coli* ESBLs at CMJAH parallels increases seen in KZN hospital obstetric departments (2015: 9.8% and 2016: 16.7%) and urban and rural community healthcare centres (11.5%).^14,15^ *Escherichia coli* ESBL rates in community-acquired UTIs are increasing globally.^17^ Whilst the CMJAH UTIs caused by *K. pneumoniae* were fewer than those as a result of *E. coli*, the *K. pneumoniae* ESBL rate is noteworthy. The pooled data for four Gauteng tertiary level antenatal care units for 2015–2019 showed ESBL rates ranging from 6% to 9% amongst the *E. coli* and *K. pneumoniae* isolates.^14^ A 2017 meta-analysis of ESBL colonisation and infection rates in African pregnant and peripartum women found a 22% prevalence of ESBLs in community urine and stool samples from Nigeria, Madagascar, South Africa and Cameroon.^18^ Ceftriaxone remains an appropriate empiric agent for pyelonephritis currently, but close monitoring of ESBL rates in the pregnant population is required.

Carbapenem-resistance amongst the Enterobacterales in hospital-acquired and healthcare-associated UTIs is well documented.^19^ The epidemiological data are lacking in this study to determine the definite origin/classification of CRE *K. pneumoniae* and *P. mirabilis* infections as community versus healthcare-associated. Determining the risk factors for ESBL and CRE UTIs in the pregnant population is an important area for further investigation. Prior antibiotic therapy, healthcare exposure, colonisation following travel to areas where multidrug-resistant organisms (MDROs) are endemic, recurrent UTIs, complicated UTIs and chronic medical conditions have been identified as risk factors for UTIs with MDROs in the general population.^17,20^

High rates of *E. coli* susceptibility to nitrofurantoin were maintained over the 6-year period. This is in keeping with local and global data.^5,6,14,16^ The multimodal mechanism of action of nitrofurantoin limits the emergence of acquired resistance.^21^ In addition, the pharmacokinetic properties of nitrofurantoin make it an ideal agent for the treatment of lower UTIs (good oral bioavailability, rapid clearance from serum and elimination largely in urine). Furthermore, nitrofurantoin has minimal impact on the gut microbiome in comparison with other antibiotics commonly used for UTIs.^22^ *Proteus* species are intrinsically resistant to nitrofurantoin.

Like nitrofurantoin, fosfomycin showed consistently high levels of activity against *E. coli* including the ESBL-producing isolates. There is limited published data for fosfomycin from other South African centres. The 2011 Lewis et al. Gauteng community-acquired UTI study reported 98.3% fosfomycin susceptibility amongst Gram-negative pathogens.^5^ Internationally fosfomycin susceptibility is also high amongst *E. coli* isolates despite use of this antimicrobial for many years.^23^ The activity of fosfomycin against Enterobacterales other than *E. coli* is not reliable. As such, CLSI and EUCAST currently only have breakpoints for *E. coli*. Advantages of oral fosfomycin include single dose administration (associated with good compliance), attainment of prolonged high urinary concentrations and minimal associated collateral damage such as dysbiosis and colonisation with MDROs.^23^ A recently published meta-analysis concluded that clinical outcomes of fosfomycin therapy for ASB and symptomatic UTIs in pregnancy are equivalent to that of other antibiotics (beta-lactams, sulphonamides, quinolones and nitrofurantoin).^24^

The high rates of ampicillin susceptibility amongst the *E. faecalis* and *S. agalactiae* isolates are in keeping with results of the other South African studies.^6,14^ Pooled data for 301 *E. faecalis* isolates from three other Gauteng tertiary ANC units, which serve a similar obstetric patient population to CMJAH (Chris Hani Baragwanath Academic Hospital, Rahima Moosa Mother and Child Hospital and Steve Biko Academic Hospital), showed a susceptibility rate of 99% to nitrofurantoin.^14^ High rates of *E. faecalis* susceptibility to nitrofurantoin have been reported internationally as well.^25,26^ Fosfomycin susceptibility testing of a subset of CMJAH 2015/2016 *E. faecalis* isolates showed high rates of susceptibility (98%).^27^ Fosfomycin also shows good activity against *E. faecalis* in the published literature.^25,28^ Whilst *E. faecalis* nitrofurantoin and fosfomycin susceptibility data were not available for this study population specifically, it is expected that these antimicrobials will provide high levels of cover for *E. faecalis* isolates from CMJAH ANC patients. However, this must be confirmed by the CMJAH Microbiology Laboratory through the implementation of routine testing of these isolates or the performance of periodic surveillance testing. Urinary tract infections caused by *S. aureus* comprised a small proportion of all infections. However, methicillin-resistant *S. aureus* (MRSA) infections were documented in 2019 and 2020. Risk factors for UTIs caused by MRSA in pregnancy must be explored. *Staphylococcus aureus* has been reported as important uropathogen in parts of Africa.^4^

The EDL-recommended agents, nitrofurantoin and fosfomycin, provide good coverage for *E. coli*, the top pathogen at CMJAH and Gauteng tertiary ANCs for which published epidemiological data are available. Based on the high rate of *E. faecalis* nitrofurantoin susceptibility reported from other Gauteng tertiary ANC populations, the use of nitrofurantoin as empiric therapy is acceptable. The lower susceptibility rates of *K. pneumoniae* to nitrofurantoin (46% in 2020) and the questionable activity of fosfomycin against this organism make these EDL recommended antibiotics less reliable for *K. pneumoniae*.As a result of the much lower proportion of UTIs caused by *K. pneumoniae* (8.9% in 2020), the current practice of empiric nitrofurantoin use at CMJAH remains appropriate. Fosfomycin use at CMJAH is generally reserved as directed therapy for nitrofurantoin-resistant uropathogens. With the greater prevalence of *K. pneumoniae* in KZN, investigation of the cumulative coverage provided by nitrofurantoin and fosfomycin in this setting is indicated. Amoxicillin-clavulanate shows good activity against the top three pathogens. However, amoxicillin-clavulanate use in pregnancy has been associated with a significant increase of necrotising enterocolitis in the neonate.^29^

Reductions in maternal and neonatal mortality through improved antenatal care and fewer prematurity-related complications in neonates are important components of the United Nations Sustainable Development Goals.^30^ Neonatal outcomes are closely related to maternal health status. Data from a Tanzanian neonatal unit demonstrated that maternal colonisation with ESBL Enterobacterales was a risk factor for neonatal colonisation and sepsis with these organisms.^31^ A German study in two neonatal units, using strain typing, showed that maternal colonising ESBL strains were related to those colonising their neonates.^32^ Efforts to optimise antimicrobial use (particularly in the context of UTIs as these are the most common infection type in this population) and reduce AMR in the obstetric patient population will positively impact neonatal outcomes too.

Data from other provinces and different levels of care, particularly primary care (although the expectation is that lower levels of resistance will be seen at this level compared to ANCs at tertiary hospitals), are required to determine the ongoing applicability of national recommendations.

Strengths of this study include the availability of recent and longitudinal data for 6 years and the *E. coli* fosfomycin data (not available from other recent South African publications). This study has a number of limitations. It is a single tertiary centre study, and the epidemiology will not be representative of that in other provinces and levels of care. The CMJAH ANC sees both walk-in patients from the community and patients referred from surrounding centres. It was not possible to separate out hospital-acquired infections from community-acquired ones. It is expected that the resistance rates found in this study setting are higher than those in primary healthcare settings. Non-speciation of 47 CoNS isolates over the 6 years may have resulted in an underestimation of *S. saprophyticus* prevalence, but these species would not constitute more than 3% of all isolates. Cefazolin susceptibility data for the Enterobacterales was not available. However, based on the cefuroxime data, cefazolin would not be an appropriate empiric agent. Nitrofurantoin and fosfomycin data for *E. faecalis* were not available. However, data from other Gauteng studies show favourable susceptibility rates to these antibiotics. For the beta-lactam antibiotics categorisation of intermediate susceptibility results as ‘resistant’ for data analysis purposes, may have resulted in underestimation of the potential activity of these antibiotics.

Consistent with other reports from the country, our study reiterates the rising rates of resistance amongst uropathogens causing UTIs in pregnant women. Ongoing surveillance and further clinical studies in this setting are necessary to determine risk factors, effect of various antimicrobial agents and outcomes for both mother and infant. We have highlighted the limited treatment agents available. Further pharmaceutical interest in development of antimicrobial agents, which are safe during pregnancy, is both imperative and urgent because these rising resistance rates could potentially threaten the progress of the UN sustainable development goals.
